# Validity and reproducibility of match-derived ratios of selected external and internal load parameters in soccer players: A simple way to monitor physical fitness?

**DOI:** 10.5114/biolsport.2023.124850

**Published:** 2023-03-06

**Authors:** Jan Schimpchen, Paulo Freitas Correia, Tim Meyer

**Affiliations:** 1 Sport Lisboa e Benfica, Human Performance Department, Lisbon, Portugal; 2 Institute of Sport and Preventive Medicine, Saarland University, Saarbrücken, Germany

**Keywords:** Heart rate, GPS, Physical performance, Lactate, Football

## Abstract

The study aim was to assess whether match-derived external-to-internal load ratios are a valid and reliable tool to measure physical fitness. Sixteen elite youth soccer players (17 ± 1 years) performed two maximal fitness tests. Subsequently, players participated in three intra-squad soccer matches in three consecutive weeks. Three GPS-based parameters of external load (total distance, PlayerLoad, high-intensity distance) were divided by three heart rate-based parameters of internal load (iTRIMP, Banister TRIMP, average percentage heart rate reserve) for the ratio calculations. Validity was established by comparing the ratios with results of the fitness tests, while between-athlete and within-athlete reliability were quantified. Most integrated load ratios were moderately-to-largely correlated with the various fitness parameters. Overall, a ratio consisting of PlayerLoad and average percentage heart rate reserve demonstrated the most consistent correlations with maximum treadmill speed (r = 0.69, P = 0.003) and the speeds associated with 4 mmol/L of blood lactate (r = 0.56, P = 0.024) and 80% of heart rate reserve (r = 0.54, P = 0.031). Most of the ratios displayed acceptable levels of reproducibility (intraclass correlation coefficient > 0.8 and coefficient of variation < 10%), with the minimal detectable change of all ratios ranging between 7.1 and 37.8%. Given their associations with physical fitness and non-invasive nature, certain external-to-internal load ratios may be used to monitor physical fitness in soccer players. However, the ratios may not be sensitive enough to detect small yet practically relevant alterations in player fitness.

## INTRODUCTION

The development and in-season maintenance of high levels of physical fitness is an important consideration for practitioners tasked with maximising the performance potential of soccer players. Ideally, accurate information on fluctuations in player fitness can be obtained regularly to tailor training interventions to the individual player needs [1]. These insights are typically derived from (maximal) laboratory- or field-based fitness tests. However, due to their exhaustive nature and the difficulty of finding suitable time points within an oftentimes congested playing schedule, it is unrealistic to expect frequent testing opportunities at the higher levels of professional soccer [2]. Given these limitations, it would seem desirable to establish alternative testing protocols which are valid, reliable, minimally invasive and require little to no extra time or effort by the players [3, 4].

Within elite soccer, training and match physical demands are commonly recorded using a host of different internal and external load monitoring tools [2]. It has been proposed that the relationship between identified markers of internal and external load may provide suitable information regarding how a player is coping with their training programme [5]. As such, under standardised and non-fatigued conditions, improved fitness would be assumed for players experiencing a lower internal load relative to a standardised external task, which may be attributed to various central and peripheral adaptations resulting in improved exercise economy, whole-body peak oxygen uptake, lactate threshold and oxygen uptake kinetics [6]. Vice versa, players with a higher internal load relative to the same external task would be suspected to have experienced negative fitness adaptations [5]. Similar conclusions may be deduced from relative changes in internal load in response to non-standardised external stimuli. Several studies have demonstrated significant correlations between external-to-internal load ratios during sport-specific, non-standardised activity and parameters of aerobic performance in soccer [7, 8], rugby [9] and hurling players [10]. Given that markers of external load have been shown to be highly variable in competition [11] and may be impacted by contextual factors [12], the integration of external and internal load into a ratio represents a rather elegant way to assess athlete fitness since the parameters necessary for calculation are typically recorded as part of the monitoring routine of elite soccer teams [13], avoiding the need for any additional ‘touch points’ with and burden on the players [14].

Akubat et al. [7, 8] investigated different combinations of integrated external-internal load ratios obtained during a soccer match-specific running simulation. The authors used five different GPS- or accelerometer-derived parameters of external load (total distance, high-intensity distance, PlayerLoad, mean metabolic power, distance covered at high metabolic power) and individualised TRIMP (iTRIMP) as the only parameter of internal load. Since heart rate (HR)-based parameters are easily and precisely measured, observing the cardiocirculatory response to exercise is frequently the preferred method of monitoring internal load in a team-sport context. In a recent study, iTRIMP was found to be the training load measure with the strongest relationship to changes in aerobic fitness over a 6-week pre-season period in elite youth soccer players [15], and therefore its inclusion in the integrated external-internal load ratios seems to be justified. However, since the calculation of iTRIMP is based on each player’s lactate profile to generate an individual exponential weighting factor for the TRIMP calculation [16], it is very context-dependent whether the necessary testing procedures can be carried out (potentially multiple times per season). It would appear worthwhile to investigate whether other more easily obtainable parameters of cardiocirculatory load might be a feasible alternative to iTRIMP in the calculation of the integrated ratios. Furthermore, since the two studies assessing the relationship between different external-internal load ratios and aerobic fitness in soccer players were based on running protocols looking to replicate typical match running demands [7, 8], it remains to be confirmed whether ratios based on actual match-derived markers of external and internal load are equally related to parameters of cardiocirculatory fitness. If so, this would potentially allow for continuous fitness monitoring based on match data alone.

When interpreting any test score, it is important to be aware of and appreciate the uncertainty associated with the measure [17]. Preferably, a test chosen for implementation should be responsive to small but relevant changes over time. As such, if match-derived external-to-internal load ratios are to be used for monitoring changes in player fitness, the ratios should be able to differentiate signal (real change) from noise (random measurement error). In this context, the minimal detectable change (MDC) and the smallest worthwhile change (SWC) [18–20] are two important test statistics which can help quantify signal (SWC) in comparison to noise (MDC). MDC refers to the smallest change that can be detected with an acceptable probability level, while SWC relates to the smallest change that can be considered important.

Therefore, the aims of this study were twofold: (1) to investigate whether different combinations of match-derived external-internal load ratios were associated with markers of aerobic fitness; and (2) to quantify the reliability of these measures across multiple time points.

## MATERIALS AND METHODS

### Participants

Twenty-one youth outfield players (17 ± 1 years, 179 ± 8 cm, 72 ± 7 kg) belonging to an elite Portuguese soccer team were recruited to participate in the study [21]. An a priori sample size calculation (G*Power, version 3.1.9.7, Heinrich Heine University Düsseldorf, Germany) indicated that 21 participants were required to have a sufficient power (> 0.8) based on the alpha selected (5%) and a large effect size (d = 0.5). This effect size was based on previous studies on this topic [7, 8]. Players were eligible to participate in the study if they had been free of injury up to four weeks prior to the beginning of the study and accustomed to the regular training routine of the team. The players and their parents were informed about the study procedures and provided their written informed consent. Performance data were anonymised to ensure complete player confidentiality. The investigation was conducted in accordance with the Declaration of Helsinki and ethical approval was received from the local ethics committee (Saarland University, approval number 20–26).

### Design and Data Collection

**FIG. 1:**
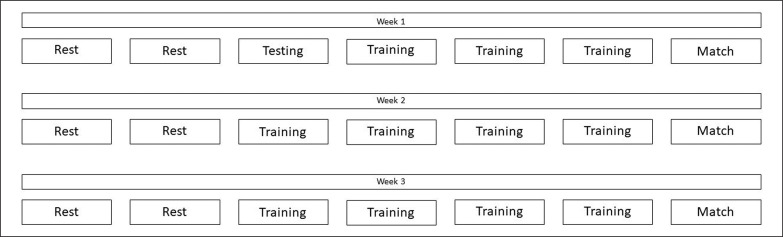
Overview of study design.

Due to local COVID-19 regulations during the study period, the participating team was not involved in official competition. However, the team had been training for 14 weeks straight before the beginning of the investigation, with the training week consisting of four training sessions from Monday to Thursday and an intra-squad, full-pitch 90 min match each Friday, followed by two days of rest. This weekly structure was largely maintained for our study purposes (Fig. 1).

This observational study began with an evaluation of the players’ cardiocirculatory fitness. Throughout the morning (8 am–12 pm) and 90 minutes after their last meal, all players performed a stepwise incremental treadmill running test with determination of capillary blood lactate and HR responses. The test was initiated at a running velocity of 8 km · h^−1^, with a step duration of 3 min, a 2 km · h^−1^ velocity increment for each successive step until volitional exhaustion occurred, with steps separated by a 1 min passive recovery period for the collection of capillary blood samples from the participants’ earlobes (Lactate Pro 2, Arkray KDK, Kyoto, Japan). HR was continuously monitored (Firstbeat Sports Sensor, Firstbeat Technologies Oy, Jyväskylä, Finland) at a sampling frequency of 0.2 Hz and exercise HR for a given velocity was defined as the mean HR over the last 30 s for each step. The treadmill gradient was set at 1% to reflect the energetic cost associated with outdoor running [22]. After the test, the data were fed into specialised software (Ergonizer, Kai Röcker, Freiburg, Germany) and the following parameters were calculated for further analysis: (1) running velocity at volitional exhaustion (Vmax) (if subjects did not complete the final stage, Vmax was calculated as: (running velocity of last completed stage) + (x/180*2 km · h^−1^), where × equals the duration of the final stage in s), (2) running velocity associated with a fixed lactate concentration of 4 mmol/L (V4 mmol), and (3) the physical working capacity (running velocity) at 80% of the participants’ individual HR reserve (V80%HR_Res_). During the late afternoon of day 1 (4 pm–6 pm) and after at least 6 h of passive recovery from the previous assessment, the players performed a Yo-Yo Intermittent Recovery level 1 test (Yo-Yo IR1). The test was executed on an artificial outdoor turf, at an ambient temperature of 15°C. The Yo-Yo IR1 has been shown to possess high levels of construct validity and may therefore be considered a good indicator of intermittent team sport-specific physical fitness [23]. The running distance associated with the last completed stage before failing to maintain the indicated running velocity was recorded as the final test result.

All training sessions throughout the study period were planned and led by the team’s coaching staff. Before the beginning of the investigation, team coaches were instructed to maintain their normal training periodisation leading up to the matches and, as such, expose the players to the regular training volumes and intensities associated with their training approach. Overall training load throughout the study protocol was kept consistent to maintain the players’ physical condition. In addition, players continued to perform two gym-based training sessions per week as per their usual training routine. These sessions took place four and two days prior to the match and consisted of full-body, multi-joint strength and power exercises (first weekly session) and exercises focused on lumbo-pelvic control, landing mechanics and balance (second weekly session).

During all matches, player movement was recorded using portable GPS devices (Catapult Vector, Melbourne, Australia). Previous studies have found 10 Hz GPS systems to be the most valid and reliable across linear and team sport simulated running. Accuracy measures of total distance and high-intensity distance displayed a coefficient of variation (CV) of between 1.9 and 10.5% compared to criterion measures [24]. After the match, all data were downloaded using the respective proprietary software (Catapult Openfield, version 3.2.0) and adequate positional data quality was verified. In accordance with previous research [25], GPS connection to at least 6 satellites and a horizontal dilution of precision (HDOP) ≤ 2 were required for data to be included. Throughout the final dataset, the number of connected satellites and HDOP were 14.7 ± 0.6 and 0.7 ± 0.1, respectively. The players’ total distance (TD) covered, the distance covered at running speeds > 18 km · h^−1^ (HID) and PlayerLoad (PL – a widely used external load metric derived from three-dimensional measures of the instantaneous rate of change of acceleration) [26] were collected as markers of external load over the course of the three matches. These three parameters were deemed relevant since they are commonly used to quantify training and match demands. HR was continuously recorded throughout the matches and the resulting cardiorespiratory intensity and load were calculated in three different ways: (1) as the average percentage of HR_Res_ (ave%HR_Res_), (2) as a Banister TRIMP score (BanTRIMP) [27], and (3) as an iTRIMP score [16]. Consequently, nine different external-to-internal load ratios were calculated and investigated.^1^

Resting HR for each player was determined one morning following two days of rest, in a quiet room with players remaining in a supine position for 5 min. Resting HR was assumed as the lowest 5 s value. Maximal HR was assumed as the highest 5 s value throughout all testing, training and match activities during the study period.

### Statistical Analysis

Unless stated otherwise, all data are presented as mean ± standard deviation (SD). The assumption of normality was verified using the Shapiro-Wilk test. Pearson’s correlation coefficients were used to quantify the relationship between external-to-internal load ratios recorded during the first match and fitness markers. The magnitude of the correlation was interpreted as follows: < 0.1, trivial; 0.1–0.29, small; 0.3–0.49, moderate; 0.5–0.69, large; 0.7–0.89, very large; 0.9–0.99, nearly perfect; and 1, perfect [28]. Between-athlete and within-athlete reliability were assessed using intraclass correlation coefficients (ICC) and CV, respectively. ICC estimates and their 90% CI were calculated based on a mean-rating (k = 3), absolute agreement, 2-way random-effects model. The following thresholds for interpretation of ICC results were used: < 0.5, low; 0.5–0.74, moderate; 0.75–0.89, high; 0.9–0.98, very high; and > 0.98, extremely high. CV was calculated by dividing the within-subject SD by the mean multiplied by 100. Furthermore, MDC at 90% CI level was calculated as 1.645 × SEM × √2 [18] and SWC was calculated using a distribution-based method as 0.2 × between-subject standard deviation [19]. Standard error of measurement (SEM) was estimated as the square root of the mean square error from the ANOVA [29]. Ratio reliability and association with fitness markers were analysed separately for first and second halves of the matches. A player entering a match at halftime was considered the same as playing the first half of the match and only players competing in at least a full half were included for analysis. Only players competing in all three matches were considered for the reliability analysis. An α-level of *P* ≤ 0.05 was accepted as statistically significant. All statistical analyses were conducted using specialised Excel spreadsheets [30].

## RESULTS

During the initial incremental treadmill test, players achieved a Vmax of 17.7 ± 1.3 km · h^−1^, at a HR of 195 ± 7 beats · min^−1^, representing 98.2 ± 1.7% of their maximal HR (HR_max_), with a maximal blood lactate concentration of 8.4 ± 2.5 mmol/l. V4 mmol was associated with a running velocity of 15.1 ± 1.4 km · h^−1^, at 93.3 ± 2.5% of HR_max_. V80%HR_Res_ occurred at 12.1 ± 1.3 km · h^−1^ and an average blood lactate concentration of 2.3 ± 0.4 mmol/l. The last completed stage of the Yo-Yo IR1 test corresponded to a running distance of 2181 ± 484 m, at 96.6 ± 2% of HR_max_.

### Relationship between load ratios and fitness markers

**FIG. 2:**
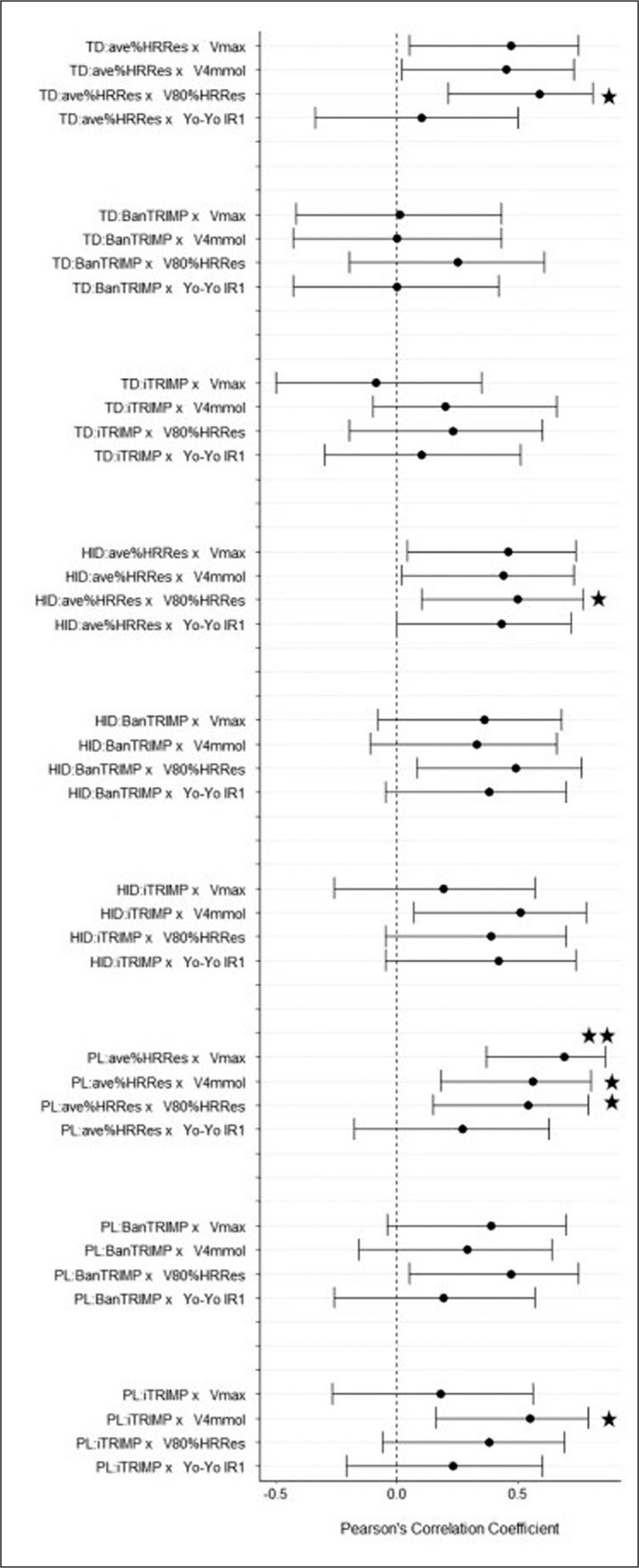
Relationship between the different external-to-internal load ratios derived during the first half of match play and selected markers of physical fitness. Note: TD – total distance; HID – high-intensity distance; PL – PlayerLoad; Vmax – velocity at volitional exhaustion; V4 mmol – velocity associated with 4 mmol/L of blood lactate; V80%HR_Res_ – velocity at 80% of heart rate reserve; Yo-Yo IR1 – Yo-Yo intermittent recovery level 1 test performance; * – P ≤ 0.05; ** – P ≤ 0.01.

**FIG. 3:**
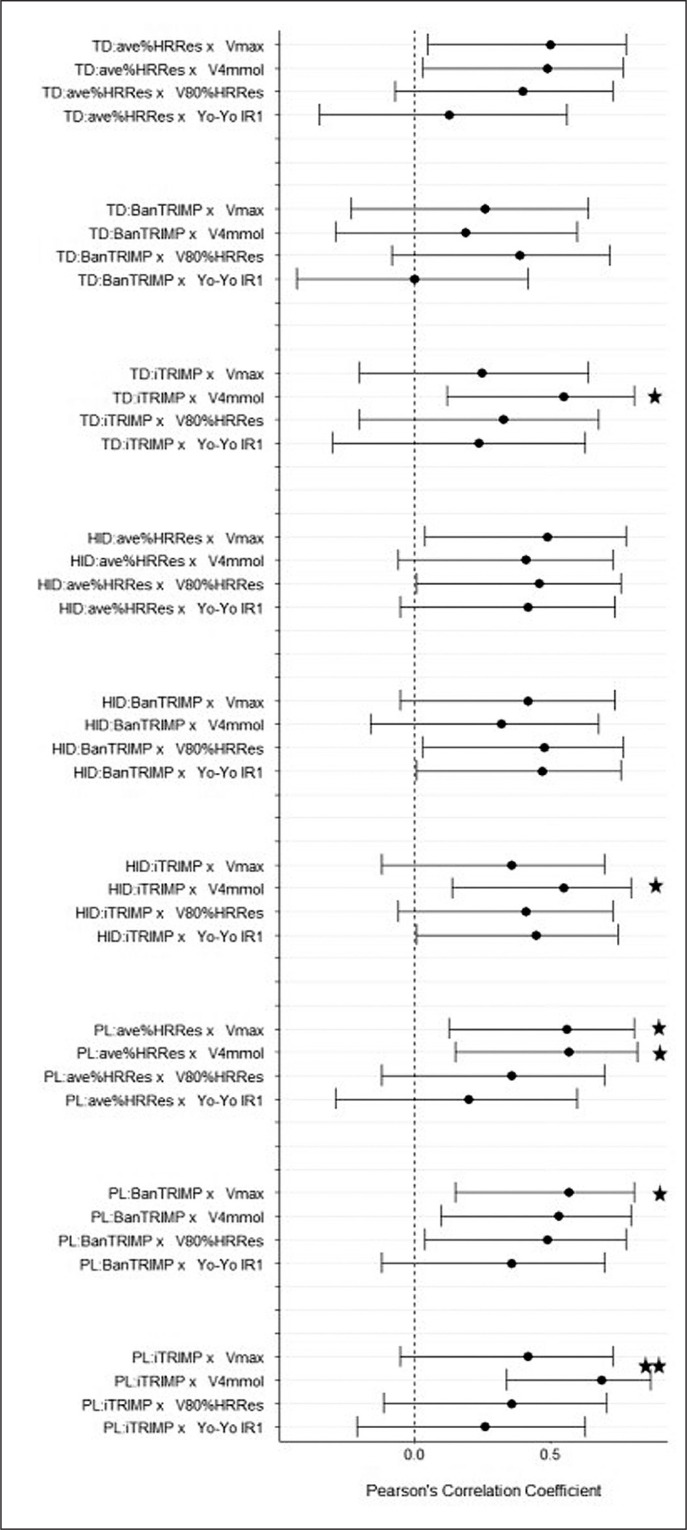
Relationship between the different external-to-internal load ratios derived during the second half of match play and selected markers of physical fitness. Note: TD – total distance; HID – high-intensity distance; PL – PlayerLoad; Vmax – velocity at volitional exhaustion; V4 mmol – velocity associated with 4 mmol/L of blood lactate; V80%HR_Res_ – velocity at 80% of heart rate reserve; Yo-Yo IR1 – Yo-Yo intermittent recovery level 1 test performance; * – P ≤ 0.05; ** – P ≤ 0.01.

Two players dropped out of the investigation during the first week of training, one due to injury and the other due to illness. Furthermore, three players were excluded from the analysis due to incomplete HR recordings during the first match. 14 of the remaining 16 players completed the entire first match, while two players only completed the first half. The relationships between the different external-to-internal load ratios and fitness markers are presented for the first and second half in Figures 2 and 3, respectively.

### Reproducibility of load ratios

The three matches were played on the same regulation size, natural grass outdoor pitch under very similar environmental conditions (humidity: 92 ± 9%, temperature: 14 ± 3ºC, wind speed: 17 ± 6 km · h^−1^). Both teams played in a 4-3-3 formation with players maintaining their tactical position through all games. Three players missed a game due to being called up to train with an older team within the club’s academy structure and one player had to be excluded from the analysis due to injury during the final match. Of the remaining 12 players, 7 completed the full 90 minutes in all three consecutive matches. Throughout the three matches, those 7 players on average covered a TD of 11,130 ± 909 m, with a HID of 1,067 ± 236 m, a PL of 1,184 ± 183 AU, at an average of 80 ± 4% of HR_Res_, with a BanTRIMP score of 226 ± 23 AU, and an iTRIMP score of 214 ± 40 AU. Between-athlete and within-athlete reproducibility of the different integrated external-to-internal load ratios is presented in Table 1.

**TABLE 1:** Between-athlete and within-athlete reproducibility of the different external-to-internal load ratios

External: Internal Ratio	Half	ICC (90% CI)	CV (90% CI)	SEM	MDC	SWC	SEM %	MDC %	SWC %
TD:iTRIMP	1^st^	0.90 (0.77; 0.96)	7.6 (6.0; 10.8)	4.5	10.5	2.4	8.1	18.8	4.3
	2^nd^	0.90 (0.8; 0.98)	8.7 (6.4; 14.5)	5.6	13.0	3.0	10.0	22.3	5.2

PL:iTRIMP	1^st^	0.78 (0.56; 0.91)	9.6 (7.6; 13.5)	0.5	1.2	0.2	9.2	21.3	3.8
	2^nd^	0.88 (0.65; 0.97)	10.7 (7.9; 18.0)	0.6	1.5	0.3	10.7	24.8	4.9

HID:iTRIMP	1^st^	0.88 (0.73; 0.95)	15.6 (12.3; 21.8)	0.9	2.2	0.5	16.3	37.8	8.0
	2^nd^	0.91 (0.73; 0.98)	15.5 (11.5; 25.9)	0.9	2.1	0.5	14.9	34.7	8.1

TD:BanTRIMP	1^st^	0.83 (0.64; 0.93)	4.8 (3.8; 6.7)	2.5	5.8	1.1	5.0	11.7	2.1
	2^nd^	0.75 (0.38; 0.93)	6.7 (5.0; 11.3)	3.7	8.6	1.1	7.4	17.1	2.3

PL:BanTRIMP	1^st^	0.70 (0.43; 0.87)	6.9 (5.5; 9.8)	0.3	0.8	0.1	6.6	15.4	2.4
	2^nd^	0.79 (0.45; 0.94)	7.9 (5.7; 13.2)	0.4	1.0	0.1	8.2	19.0	2.8

HID:BanTRIMP	1^st^	0.80 (0.59;0.92)	14.5 (11.5; 20.4)	0.8	1.8	0.3	15.2	35.4	6.0
	2^nd^	0.79 (0.45; 0.94)	14.7 (10.9; 24.6)	0.7	1.5	0.3	12.6	29.3	5.3

TD:ave%HR_Res_	1^st^	0.83 (0.65; 0.93)	2.8 (2.2; 4.0)	2.1	4.9	0.9	3.1	7.1	1.3
	2^nd^	0.89 (0.67;0.97)	3.3 (2.5; 5.6)	2.6	6.1	1.1	3.8	8.9	1.6

PL:ave%HR_Res_	1^st^	0.87 (0.71; 0.95)	5.6 (4.4; 8.0)	0.4	0.9	0.2	5.6	13.1	2.8
	2^nd^	0.95 (0.85; 0.99)	4.2 (3.1; 7.0)	0.4	0.8	0.2	4.9	11.4	2.9

HID:ave%HR_Res_	1^st^	0.75 (0.50; 0.90)	15.0 (11.8; 21.1)	1.1	2.6	0.4	16.1	37.6	5.5
	2^nd^	0.65 (0.22; 0.9)	16.2 (12.1; 27.2)	0.9	2.1	0.4	12.9	29.9	4.9

Note: ICC – intraclass correlation coefficient; CI – confidence interval; CV – coefficient of variation; SEM – standard error of measurement; MDC – minimum detectable change; SWC – smallest worthwhile change; TD – total distance; PL – PlayerLoad^TM^; HID – high-intensity distance; iTRIMP – individualized TRIMP; BanTRIMP – Banister TRIMP; ave%HR_Res_ – average percentage of heart rate reserve

## DISCUSSION

The present study sought to establish the association of different combinations of match-derived external-to-internal load ratios with various submaximal and maximal fitness parameters and to quantify their reproducibility across three time points. The main findings were that (i) while most combinations of integrated load ratios were moderately-to-largely correlated with the various fitness parameters at a single time point, these results must be interpreted cautiously due to the rather wide confidence intervals; (ii) the combination of PL as an external load parameter and ave%HR_Res_ as an internal load parameter demonstrated the most consistent associations with different fitness markers in comparison to other load ratios; (iii) ave%HR_Res_ can be used as a simpler internal load alternative to iTRIMP for the ratio calculations; (iv) combinations involving HID showed reduced within-athlete reproducibility compared to combinations involving TD and PL as external load parameters; and (v) none of the ratios may be sensitive enough to detect small but relevant changes in athlete fitness.

In comparison to previous studies [7, 8], the relationships between different combinations of external-to-internal load ratios and markers of physical fitness found in the current study were less strong and less consistent. This may be partially explained by the use of actual match-derived external and internal load data as opposed to data derived from soccer match running simulations. While the results of simulations of physical match demand may be associated with isolated markers of physical fitness and may reproduce certain aspects of match running performance [31], these protocols miss important physical actions and their impact on internal load, specifically in relation to short but intense accelerations and decelerations, changes of directions, dives, jumps or tackles [32]. Furthermore, the cognitive stressors associated with ball movement activities and the tactical reading of the game cannot be adequately replicated by match running simulations. In fact, it has been suggested that team sport athletes may experience mental fatigue due to prolonged and demanding match-related cognitive activity and that mental fatigue may induce reductions in physical performance during intermittent team sport activity [33]. It is important to point out that the observed reduction in physical performance in those studies was most likely due to an increased perception of effort rather than cardiovascular or metabolic mechanisms. Therefore, it seems fair to speculate that match-derived external-to-internal load ratios may be influenced not only by physical, but also by mental requirements. As such, comparably weaker associations between these load ratios and laboratory-derived markers of physical fitness are not necessarily surprising. This should not, however, automatically be interpreted as a potential weakness of the load ratios. While isolated fitness tests can more accurately determine changes in the physiological profile of a player, integrated load ratios may still be able to more holistically capture a player’s ability to withstand competition-specific demands.

Apart from match-derived load ratios, another promising approach may be the integration of external and internal load data derived from standardised training drills. Training drills can be designed to possess fewer degrees of freedom compared to match play and may therefore correlate better with markers of fitness tests. In fact, Taylor et al. [9] demonstrated that integrated load ratios derived from a sprint interval training drill (6 × 6-second sprint with 54 seconds recovery) and from a small-sided game (6 vs. 6, 10 minutes on a 39 × 51 m pitch) showed large-to-very large associations with submaximal fitness markers and acceptable reliability in academy rugby union players. While similar results might be expected for soccer players and, therefore, potentially represent an interesting alternative to match-derived data, we want to highlight that no training drill can totally replicate all physical, technical, tactical, and cognitive demands that players experience during actual match play. As such, a certain trade-off between standardization and specificity should be expected.

Previous studies have already investigated different external load parameters to be included in the integrated load ratio calculations [7–10]. However, iTRIMP was the sole parameter of internal load to be examined. The results of the present study indicate for the first time that ave%HR_Res_ can be used as a less complex alternative to iTRIMP, facilitating an easier use of external-to-internal load ratios as a marker of physical fitness. While these findings seemingly favour a simpler approach with regards to the internal load parameter, a reduction of the chaotic nature of professional soccer matches into a single locomotive parameter might be overly simplistic. Lacome et al. [34] argue, for instance, that a more individualised approach taking into account player-specific combinations of external load variables may be more suitable to detect alterations in player-specific fitness. While their approach largely correlates with the results of a submaximal running test, the magnitude in correlation is not substantially larger than the best performing ratio in the present study. Therefore, it appears that more sophisticated approaches with regards to both external and internal load calculations do not necessarily outperform integrated ratios consisting of more easily derived parameters. Also, the attractiveness of easy-to-use indicators with high face value must be emphasised.

The present study was the first to quantify the between-athlete reliability of integrated load ratios, generally demonstrating high-to-very high reproducibility. With regards to within-athlete reliability, one prior study examined several external-to-internal load ratios during three different training drills (continuous shuttle run, sprint interval training, small-sided game) in academy rugby union players [9] and found the CV of the ratios to range between 7.1 and 36.3%. Higher variability was found for ratios involving high-speed running as an external load parameter, which was in line with the present study. Given that match-to-match variability in high-speed running has been shown to be high among professional soccer players [11], it seems likely that this variability carries over into the integrated load ratios. As such, it is not advised to use these specific ratios as indicators of aerobic fitness. In contrast, ratios involving TD and PL as external load parameters showed reduced variability. One possible explanation may be that TD and PL are more volume-based parameters and, therefore, are not as affected by match-to-match variability as more intensity-based parameters such as HID. Nevertheless, an analysis of MDC and SWC revealed that none of the ratios may be sensitive enough to detect small but practically meaningful changes in athlete fitness. This may not be entirely surprising given that the ratios entail the variability specific to both the external [11, 35] and the internal load [36] parameters. As such, for an integrated load ratio consisting of PL and ave%HR_Res_, only changes greater than 11–13% can be confidently assumed to be greater than the normal day-to-day variability, while changes greater than 3% may already be practically meaningful. It needs to be pointed out, however, that due to the way MDC and SWC are calculated and the probabilities of finding the true value associated with both approaches (90% confidence for MDC vs. 16% confidence for SWC), readers should not compare SWC and MDC. Instead, the values provided by the present study should be critically evaluated and interpreted specifically to a given context.

Previous research on match-derived external-to-internal load ratios has shown that there may be substantial within-athlete ratio differences between the first and second halves of a match [37], indicating that – as the match goes on – external output decreases disproportionally in relation to internal load due to accumulated fatigue. Taking this finding into account, first and second halves were analysed separately to investigate any potential differences with regards to ratio validity and reproducibility. The results showed, however, that there were no clear and consistent between-half differences, indicating that fatigue does not seem to impact these ratio properties and that periods as short as a single half can be used to calculate and interpret changes in integrated load ratios.

There are limitations to this study that readers should be aware of. Given that the ratios are based on match-derived external and internal load data, they can only be calculated for players participating in the match but not for the rest of the squad. While it is fair to argue that the fitness of starters and substitutes is more relevant to team success than the fitness of fringe players, an alternative perspective would be that changes in fitness in those players might be more likely due to a lack of regular playing time and therefore require closer attention. Furthermore, the present study established load ratio validity at a single time point. While the between-athlete comparison is an important element of a given test, analyses of within-athlete changes over time are likely the more pertinent issue for athlete monitoring purposes and should be evaluated in the future. Thirdly, both maximal fitness assessments were performed on the same day. While a passive rest period of at least 6 h between the two tests was ensured, it is possible that results of the Yo-Yo IR1 test may have been negatively influenced by the earlier treadmill test. Fourthly, it has been pointed out previously that there may be statistical problems associated with the use of ratios. In certain circumstances, ratios might not be scaling consistently across a range of measured values of the denominator, indicating that the ratio would provide biased estimates at the lower and higher ends of the range [38]. Finally, the relatively wide confidence intervals indicate that a larger sample size is required to be more confident about the results of this study.

## CONCLUSIONS

In conclusion, the findings of this study indicate that certain match-derived external-to-internal load ratios are largely correlated with submaximal and maximal markers of cardiorespiratory fitness, with a combination of PL and ave%HR_Res_ demonstrating the most consistent associations. Practitioners looking to use integrated ratios to monitor changes in fitness in soccer players need to be aware of the uncertainty associated with the ratios, however. In the case of PL:ave%HR_Res_, only changes in excess of 11.4–13.1% can be confidently assumed to represent a true change. Hence, the ratios may not be sensitive enough to detect small yet practically relevant alterations in player fitness.

## Footnotes

1 It must be pointed out that ave%HR_Res_ represents internal intensity rather than internal load. As such, ratios involving this parameter should be more correctly classified as load-intensity ratios.
